# Engineering Protease-Resistant Peptides via Non-Canonical Amino Acids: Design Strategies and Biosynthetic Advances

**DOI:** 10.3390/bioengineering13070767

**Published:** 2026-06-30

**Authors:** Chen Deng, Zhongpeng Fan, Yangyang Xu, Miaomiao Cao, Jie Liao, Meng Meng

**Affiliations:** 1 International Institute of Synthetic Biology—Canada, Toronto, ON L3R 0B8, Canada; 2 Norman Bethune International Institute of Canada for Healthcare Innovation, Toronto, ON L3R 0B8, Canada; 3 State Key Laboratory of Food Nutrition and Safety, Key Laboratory of Food Nutrition and Safety, Ministry of Education, College of Food Engineering and Biotechnology, Tianjin University of Science and Technology, Tianjin 300457, China

**Keywords:** non-canonical amino acids, protease-resistant peptides, peptide therapeutics, genetic code expansion, synthetic biology, metabolic engineering

## Abstract

Peptide therapeutics offer high target selectivity and low toxicity, but their clinical utility remains constrained by rapid proteolysis in vivo and negligible oral bioavailability. Incorporating non-canonical amino acids (ncAAs) provides a robust molecular engineering framework to overcome these pharmacokinetic bottlenecks. This review analyzes the structural and biophysical design rules of ncAA-mediated peptide stabilization, categorizing them into side-chain steric shielding, backbone conformational constraint, and stereochemical evasion of L-specific proteases. We systematically evaluate the biosynthetic milestones enabling this field, focusing on engineered orthogonal translation systems (tRNA/synthetase pairs, orthogonal ribosomes, quadruplet codons) and metabolic engineering strategies that supply fluorinated and other ncAA precursors de novo. Furthermore, we examine the translation of these technologies into clinical candidates (e.g., modified antimicrobial peptides, antibody–drug conjugates, and PROTACs) and identify scaling, immunogenicity, and computational modeling as key bottlenecks. This review serves as a technical reference for designing next-generation, hyper-stable peptide therapeutics.

## 1. Introduction

Peptides bridge the gap between small-molecule drugs and large biologics, offering superior target selectivity and low off-target toxicity. Despite these advantages, natural L-α-peptides are highly vulnerable to enzymatic hydrolysis by endogenous exopeptidases and endopeptidases, and have been widely applied in the treatment of diabetes, cancer, infectious diseases, and other conditions [1,2]. However, peptide drugs face numerous challenges in practical applications, with rapid degradation in vivo being one of the most critical issues. Natural peptides are readily recognized and hydrolyzed by various proteases in the body, resulting in short drug half-life and low oral bioavailability, which severely limits the clinical efficacy and application scope of peptide drugs [3].

To overcome this challenge, researchers have developed various chemical modification strategies to enhance the protease resistance of peptides. Among these, the incorporation of non-canonical amino acids (ncAAs) represents an effective approach. NcAAs refer to all amino acid analogs beyond the 20 standard amino acids, which can be introduced into peptide backbones through chemical synthesis or biosynthesis, thereby conferring novel physicochemical properties and biological activities to peptides [4,5]. In recent years, with the rapid development of genetic code expansion (GCE) technology, researchers have been able to achieve site-specific incorporation of ncAAs in living cells by engineering the cellular translation machinery, providing unprecedented precision and flexibility for designing protease-resistant peptides [6,7].

While chemical peptide synthesis (such as solid-phase peptide synthesis, SPPS) remains the standard for short, linear peptides, genetic code expansion (GCE) provides unique advantages for peptide engineering that cannot be achieved chemically. Specifically, GCE allows for: (1) the construction of massive, genetically encoded peptide libraries (up to 10^13^ unique sequences) for high-throughput in vitro selection methods such as mRNA display and ribosome display; (2) the site-specific incorporation of non-canonical amino acids (ncAAs) into longer, structurally complex polypeptides or peptide-protein fusions where chemical coupling yields drop precipitously; and (3) the development of self-assembling host-based cell factories that biosynthesize modified peptides, bypassing the environmental burden and toxic solvents associated with chemical synthesis.

This review aims to systematically summarize the latest research progress in constructing protease-resistant peptides using non-canonical amino acids. We begin by introducing the classification and characteristics of ncAAs, detailing the structural features of various ncAAs and their mechanisms in enhancing peptide stability. Subsequently, we elaborate on design strategies for protease-resistant peptides, including cyclization, terminal protection, backbone modification, and stereochemical stabilization. We then focus on discussing the development of genetic code expansion technologies and their applications in peptide drug discovery, including orthogonal ribosome systems, quadruplet codon strategies, and novel approaches without host genome modifications. Additionally, we introduce biosynthetic and metabolic engineering methods for producing fluorinated amino acids and other ncAAs, as well as the latest progress in clinical translation. Finally, we summarize the current technical bottlenecks and prospects for future development.

## 2. Design Strategies for Protease-Resistant Peptides

**Figure 1:**
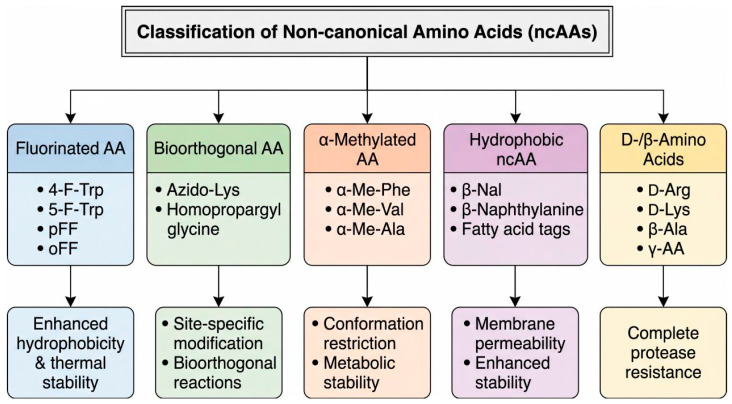
Classification and functional attributes of non-canonical amino acids (ncAAs). Schematic overview of the major classes of ncAAs employed in protein engineering and chemical biology. The classification is divided into five primary categories based on chemical structure and side-chain properties: fluorinated amino acids, bio-orthogonal amino acids, α-methylated amino acids, hydrophobic ncAAs, and D-/β-amino acids. For each category, representative examples are listed (middle tier), and the resulting physicochemical or biological advantages conferred upon the peptide or protein scaffold are highlighted (bottom tier). These modifications enable the fine-tuning of properties such as thermal stability, metabolic half-life, and site-specific reactivity, which are often unattainable using the 20 standard canonical amino acids. Abbreviations: 4-F-Trp, 4-fluorotryptophan; 5-F-Trp, 5-fluorotryptophan; pFF, para-fluorophenylalanine; oFF, ortho-fluorophenylalanine; β-Nal, β-naphthylalanine; α-Me, α-methylated.

NcAAs are diverse and can be classified into several categories based on their structural features and chemical properties (Figure 1). Table 1 provides a detailed introduction to several categories of ncAAs closely related to protease-resistant peptide design.

**Figure 2:**
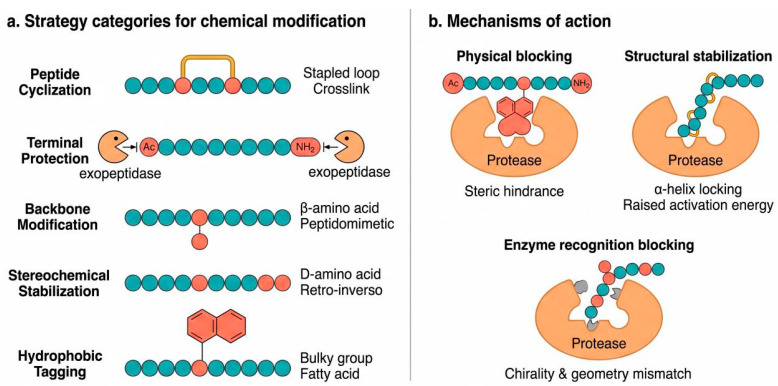
Chemical strategies and molecular mechanisms for designing protease-resistant peptides. (**a**) Five primary chemical modification approaches to enhance peptide proteolytic stability. Strategies include peptide cyclization (e.g., stapled loops or crosslinking) to restrict conformational freedom; terminal protection via N-terminal acetylation (Ac) and C-terminal amidation (NH2) to block exopeptidase access; backbone modification (e.g., β-amino acids) to disrupt active-site fit; stereochemical stabilization using D-amino acids to bypass L-specific recognition; and hydrophobic tagging with bulky groups to provide steric shielding. (**b**) Biophysical mechanisms of protease evasion. The modifications confer stability through physical blocking via steric hindrance, structural stabilization by locking secondary motifs (such as α-helices) to raise the activation energy for hydrolysis, or enzyme recognition blocking via chirality and geometry mismatches that prevent target engagement by catalytic residues.

A primary challenge in the development of peptide-based drugs is their inherent susceptibility to rapid degradation by endogenous proteases and peptidases. While peptides offer high specificity and low toxicity, the labile nature of the peptide bond often results in poor bioavailability and a short metabolic half-life. To address these pharmacokinetic limitations, a diverse toolkit of chemical engineering strategies has been developed to shield the peptide scaffold from enzymatic attack. These approaches focus on masking vulnerable termini, constraining the peptide backbone to prevent enzyme active-site entry, or altering the chemical identity of the amino acid residues to disrupt molecular recognition by L-specific proteases. By strategically implementing these modifications, the structural integrity of the peptide can be maintained within the complex proteolytic environment of the human body (Figure 2).

The chemical modifications illustrated in Figure 2 operate through three primary mechanisms of action to shield the peptide scaffold:

Steric Hindrance and Physical Shielding (Physical blocking): Modifications such as peptide cyclization (head-to-tail, side-chain crosslinking, or thioether stapling) and hydrophobic tagging (e.g., N-terminal β-naphthylalanine or lipid conjugation) physically block proteases from accessing the peptide backbone. Cyclization constrains the peptide into a rigid conformation, rendering it unable to adopt the extended, flexible conformation required to fit into the active site of most proteases. Hydrophobic tagging provides steric bulk that shields adjacent amide bonds.

Conformational Stabilization (Structural stabilization): Incorporating α-methylated amino acids (e.g., Aib) or hydrocarbon staples locks the peptide backbone into stable secondary structures, such as α-helices. Because proteases typically require the substrate to unfold or adopt a specific transition state during cleavage, stabilizing the native folded state significantly increases the thermodynamic barrier for proteolysis.

Disruption of Enzymatic Recognition (Enzyme recognition blocking): Most mammalian proteases are highly stereospecific and evolved to recognize L-amino acid residues linked by standard α-peptide bonds. Stereochemical stabilization (e.g., substituting D-amino acids or using retro-inverso designs) and backbone modification (e.g., β-amino acids or urea linkages) alter the spacing, chirality, and electrostatic characteristics of the scissile bond. This prevents the formation of key hydrogen bonds and pocket fit in the enzyme’s binding cleft, rendering the peptide completely refractory to enzymatic cleavage.

### 2.1. Peptide Cyclization

Peptide cyclization is a classical strategy for enhancing peptide stability and protease resistance. Cyclization can restrict the conformational freedom of peptides, making them more difficult for proteases to recognize and hydrolyze [34]. Based on cyclization methods, peptide cyclization can be classified into head-to-tail cyclization, side-chain crosslinking (bridging), and thioether stapling.

Head-to-tail cyclization is the simplest cyclization method, forming cyclic structures by connecting the *N*-terminus and *C*-terminus of peptides [35]. This can significantly improve resistance to aminopeptidases and carboxypeptidases. Side-chain crosslinking stabilizes peptide conformation through covalent bonds between side chains, including disulfide cyclization, click chemistry cyclization, and others [36]. Thioether stapling is a strategy that stabilizes α-helical structures by introducing intramolecular thioether bonds, simultaneously enhancing peptide stability and cell penetration capability [37].

Iskandar et al. [38] used mRNA display technology to screen cyclic peptides containing non-canonical amino acids. By employing a promiscuous orthogonal aminoacyl-tRNA synthetase (ORS) to incorporate ncAAs into peptides, they achieved pyridine-thiazoline (pyr-thn) macrocyclization. This method successfully identified a high-affinity cyclic peptide against the deubiquitinase USP15, which exhibited good selectivity for USP15 and its homologs over other ubiquitin-specific proteases.

### 2.2. Terminal Protection Strategies

The *N*-terminus and *C*-terminus of peptides are primary sites for protease recognition and cleavage. Chemical modifications of peptide termini can effectively prevent access by aminopeptidases and carboxypeptidases, thereby extending the in vivo half-life of peptides [39,40]. Common terminal protection strategies include N-terminal acetylation, C-terminal amidation, and incorporating ncAAs as protective groups. N-terminal pyroglutamation (pGlu) is a common natural modification that provides some degree of protease protection. In the aforementioned μ-CnIIIC study, researchers replaced the N-terminal pyroglutamic acid with D-amino acids, which not only improved peptide stability but also enhanced binding affinity to the target [30].

### 2.3. Backbone Modification

Backbone modification is a strategy to enhance stability by altering the main chain structure of peptides, primarily including β-amino acids and peptidomimetics [41,42]. The introduction of β-amino acids can significantly increase protease resistance because β-peptide bonds cannot be recognized and hydrolyzed by conventional proteases. β-Peptides also possess unique hydrolytic stability and cell penetration capability, and have been applied in the development of various drugs. Peptidomimetics are molecules that mimic the structure of natural peptides but possess different chemical backbones. Peptidomimetics can enhance resistance to protease degradation by introducing non-natural bonds (such as amide bonds, alkene bonds, etc.) [43].

### 2.4. Stereochemical Stabilization

Stereochemical stabilization is a strategy to enhance stability by altering peptide chirality, primarily including all-D-amino acid peptides and retro-inverso peptides [44]. All-D-amino acid peptides composed of D-amino acids are completely resistant to conventional L-specific proteases. However, all-D-amino acid peptides may lose target binding ability or produce different biological effects. Partial D-amino acid substitution can enhance stability while maintaining some biological activity [31]. Retro-inverso peptides are peptides synthesized by completely reversing the sequence of natural L-peptides and using D-amino acids. This strategy can maintain similar three-dimensional structures to parent peptides while significantly enhancing protease resistance [45]. The systematic review by Lucana et al. [3] demonstrated that enantio/retro-enantio isomerization can significantly improve the efficiency of targeting peptides and cell-penetrating peptides, especially for drug delivery to the brain.

### 2.5. Hydrophobic Tagging Enhancement

Hydrophobic tagging enhancement is a strategy to improve stability and biological activity by introducing hydrophobic groups at peptide termini [46]. As mentioned earlier, the β-naphthylalanine-tagged antimicrobial peptide N1 exhibited significantly enhanced stability and therapeutic potential. Besides β-naphthylalanine, other hydrophobic tags such as fatty acid chains and aromatic residues are also used to enhance peptide stability. Studies have shown that appropriate terminal hydrophobic modifications provide new avenues for developing highly stable peptide-based antibacterial biomaterials [27].

## 3. Biosynthetic and Metabolic Engineering Advances

**Figure 3:**
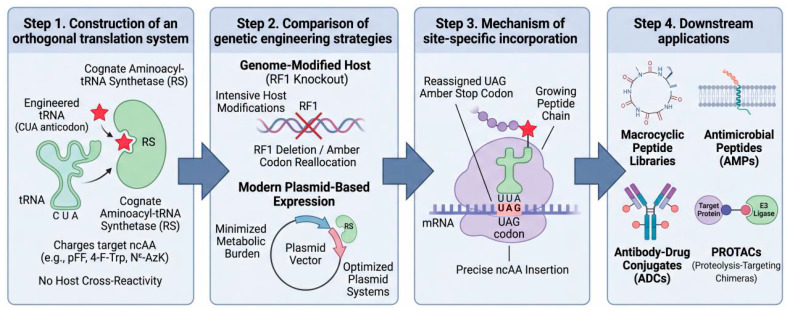
Workflow for genetic code expansion (GCE) and site-specific non-canonical amino acid (ncAA) incorporation. Schematic pipeline illustrating the progression from orthogonal system design to therapeutic application. Step 1: Construction of an orthogonal translation system. An engineered suppressor tRNA (typically with a CUA anticodon) and its cognate aminoacyl-tRNA synthetase (RS) are developed to selectively charge a target ncAA (for example, pFF, 4-F-Trp or Nε-AzK) without cross-reactivity with the host translation machinery. Step 2: Comparison of genetic engineering strategies. Traditional intensive host genome modifications, such as release factor 1 (RF1) deletion and amber codon reallocation, are contrasted with modern plasmid-based expression systems optimized to minimize metabolic burden. Step 3: Mechanism of site-specific incorporation. The engineered aminoacyl-tRNA recognizes the reassigned UAG amber stop codon on the mRNA during ribosomal translation, enabling precise insertion of the ncAA into the nascent polypeptide chain. Step 4: Downstream therapeutic applications. The modular GCE platform facilitates the development of macrocyclic peptide libraries, stable antimicrobial peptides (AMPs), site-specific antibody–drug conjugates (ADCs) and proteolysis-targeting chimeras (PROTACs).

While traditional chemical synthesis allows for the modification of small peptides, the site-specific introduction of ncAAs into larger, complex proteins and recombinant peptide libraries requires a more robust biological framework. Genetic code expansion (GCE) technology has emerged as a transformative approach to meet this need, allowing the “reprogramming” of the translational machinery to incorporate chemical moieties beyond the 20 standard amino acids. By employing orthogonal aminoacyl-tRNA synthetase (RS)/tRNA pairs—engineered to operate independently of the host cell’s endogenous genetic code—researchers can direct the incorporation of ncAAs at specific positions defined by reassigned stop codons. Modern GCE workflows have evolved from complex, genome-wide alterations toward streamlined, plasmid-based systems that minimize metabolic burden while maximizing incorporation efficiency. This technological pipeline now supports the high-throughput generation of sophisticated molecular architectures, ranging from site-specific antibody–drug conjugates to macrocyclic peptide libraries with enhanced therapeutic potential (Figure 3).

### 3.1. Orthogonal Ribosome Systems

Genetic code expansion (GCE) is a technology that incorporates ncAAs at specific codon positions by engineering the cellular translation machinery. The core of this technology is establishing an orthogonal aminoacyl-tRNA synthetase (RS)/tRNA pair that can recognize ncAAs and deliver them to the ribosome for insertion at specific codons [47,48]. The most commonly used orthogonal system is based on the pyrrolysyl-tRNA synthetase (MaPylRS) from the methanogenic archaeon Methanomethylophilus alvus and its tRNA (PyltRNA) [49]. Pyrrolysine (Pyl) is the largest known amino acid in nature, originally discovered in methyltransferases of methanogenic microorganisms. The MaPylRS/tRNA system has been successfully used to incorporate hundreds of different ncAAs in various organisms [50,51].

Alexander et al. [49] provided a detailed protocol for selecting ncAA-specific aminoacyl-tRNA synthetase/tRNA pairs from a MaPylRS active site mutant library to achieve genetic encoding of ncAAs. This protocol includes four main parts: (1) preparing the library and creating necessary cell lines; (2) selecting functional synthetases that incorporate ncAAs while selecting against synthetases that incorporate canonical amino acids; (3) three fluorescence-based detection methods to evaluate the efficiency and fidelity of surviving synthetases in incorporating the target ncAA; and (4) characterizing top hits to identify the best candidates for applications. The selection process takes approximately 30–50 days depending on preparation needs.

The review by Goettig et al. [4] systematically summarized the applications of ncAAs in protease structure and function analysis. They noted that many ncAAs can confer protease resistance to peptides, making them potential protease inhibitors and tools for substrate optimization. Other applications include in vitro and in vivo enzyme kinetics, molecular interaction studies, and bioimaging research.

### 3.2. Quadruplet Codon Strategies and Multiple ncAA Incorporation

Traditional genetic code expansion typically uses stop codons (such as UAG) as encoding sites for ncAAs. However, the use of a single stop codon limits the number of ncAAs that can be simultaneously incorporated. The quadruplet codon strategy significantly increases the number of simultaneously incorporable ncAAs by using four nucleotides to encode one amino acid [52,53].

Costello et al. [54] developed an efficient genetic code expansion method without host genome modifications. The researchers identified codon usage as a previously unrecognized contributor to efficient ncAA incorporation. Using conventional E. coli strains with native ribosomes, they developed a plasmid-based codon compression strategy that minimizes context dependence and improves ncAA incorporation at quadruplet codons. This strategy is compatible with all known genetic code expansion resources, enabling the identification of 12 mutually orthogonal tRNA-synthetase pairs. Based on this, they evolved and optimized five synthetase pairs to incorporate a broad repertoire of ncAAs at orthogonal quadruplet codons. Finally, they extended these resources to an in vivo biosynthesis platform that can readily create more than 100 novel macrocyclic peptides bearing up to three unique ncAAs.

### 3.3. Novel Strategies Without Host Genome Modifications

Traditional genetic code expansion technologies typically require genome modifications of host cells, including deleting endogenous stop codons and/or orthogonal tRNA genes to reduce readthrough and misreading. Such genome modifications are time-consuming and may affect cell growth. A recent 2025 Nature Biotechnology study proposed a novel strategy without host genome modifications [54]. The core of this strategy is to achieve efficient ncAA incorporation without using standard stop codons by optimizing codon usage and tRNA expression levels. This approach not only simplifies the construction of engineered cells but also avoids potential cytotoxicity from genome modifications. This technology has been successfully applied to create macrocyclic peptide libraries containing multiple ncAAs, demonstrating its tremendous potential in peptide drug discovery.

### 3.4. mRNA Display and In Vitro Selection Systems

mRNA display is a powerful in vitro peptide screening technology that can rapidly identify peptide ligands with therapeutic potential. By physically linking peptides to their encoding mRNA, large peptide libraries can be subjected to multiple rounds of screening to obtain high-affinity binders [55,56]. Iskandar et al. [38] demonstrated that promiscuous orthogonal aminoacyl-tRNA synthetases can incorporate ncAAs in in vitro translation (IVT) and mRNA display. They used ORS-CNF-RS to incorporate ncAAs at amber codons, including the novel substrate cyanopyridylalanine (CNpyrA), enabling pyridine-thiazoline (pyr-thn) macrocyclization in mRNA display. This work from the Bowers research group exemplifies how promiscuous synthetases can expand side-chain diversity and provide structural novelty in mRNA display libraries through heterocycle-forming macrocyclization.

### 3.5. Directed Evolution and Protein Stability

The combination of genetic code expansion technology and directed evolution methods provides powerful tools for developing proteins with novel functions. By performing mutation screening at ncAA incorporation sites, protein variants with enhanced stability, activity, or functional properties can be obtained [57]. The review by Birch-Price et al. [58] provided a detailed introduction to the applications of ncAAs in biocatalysis. They noted that genetic code reprogramming provides new insights into enzyme mechanisms by enabling the introduction of new spectroscopic probes and targeted replacement of individual atoms or functional groups. ncAAs can also be used to develop engineered biocatalysts with improved activity, selectivity, and stability, as well as enzymes with artificial regulatory elements responsive to external stimuli.

### 3.6. Biosynthetic and Metabolic Engineering Approaches

**Figure 4:**
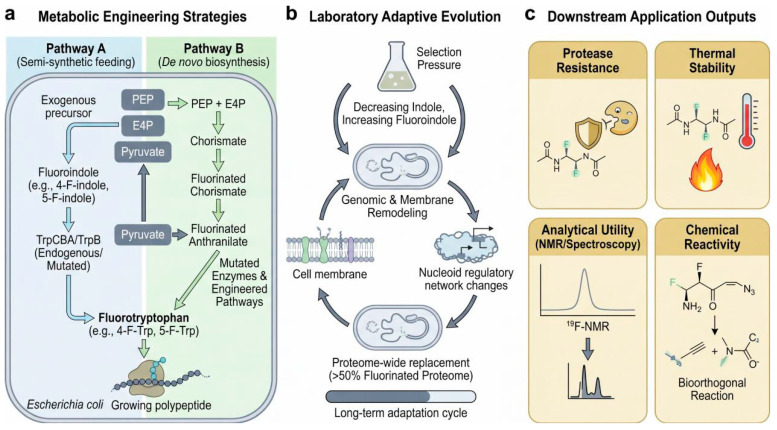
Metabolic engineering strategies and adaptive evolution for the biosynthesis and proteome-wide incorporation of fluorinated amino acids. Schematic representation of engineered pathways in Escherichia coli designed to transition from canonical metabolism to fluorinated proteome synthesis. (**a**) Comparison of biosynthetic routes. In semi-synthetic feeding (Pathway A, left), exogenous fluorinated precursors (such as 4- or 5-fluoroindole) are converted to fluorotryptophan analogues by endogenous or mutated tryptophan synthase (TrpCBA/TrpB). In de novo biosynthesis (Pathway B, right), engineered pathways synthesize fluorinated amino acids directly from central carbon metabolites (phosphoenolpyruvate (PEP), erythrose-4-phosphate (E4P), and pyruvate) via fluorinated chorismate and anthranilate intermediates. (**b**) Laboratory adaptive evolution. Tryptophan-auxotrophic E. coli undergo a long-term adaptation cycle under selective pressure (decreasing indole and increasing fluoroindole concentrations), which drives genomic and membrane remodeling to achieve extensive proteome-wide replacement (>50% fluorinated proteome). (**c**) Downstream application outputs. The incorporation of fluorinated amino acids imparts enhanced physicochemical properties to the resulting biomolecules, including superior protease resistance, increased thermal stability, distinct analytical utility (such as ^19^F-NMR spectroscopy), and novel bio-orthogonal chemical reactivity.

The incorporation of fluorine—a privileged atom in medicinal chemistry—into the biological scaffold offers a unique avenue for modulating the hydrophobicity, thermal stability, and metabolic half-life of peptides and proteins. However, achieving global, proteome-wide fluorination requires a transition from simple site-specific modification to comprehensive metabolic reprogramming of the host organism. By engineering *Escherichia coli* to bypass canonical biosynthetic constraints, researchers can implement two primary strategies: the exogenous supplementation of fluorinated precursors that hijack existing enzymatic pathways, or the more sophisticated _ biosynthesis of fluorinated amino acids from central carbon metabolites (Figure 4). When coupled with laboratory-directed adaptive evolution, these engineered strains can be “trained” to accept high levels of non-canonical residues, leading to massive proteome remodeling and membrane adaptation. This systems-level engineering approach ultimately yields a generation of biomolecules with superior physicochemical properties, including enhanced protease resistance and thermal robustness, which are otherwise unattainable in natural biological systems.

### 3.7. Metabolic Adaptation of Fluorinated Amino Acids in E. coli

Biosynthesis of fluorinated amino acids requires host cells to possess the capability to metabolically utilize fluorinated precursors. The systematic study by Agostini et al. [10] revealed the metabolic adaptation mechanisms of E. coli during long-term exposure to fluorinated amino acids. Through long-term screening, researchers exposed tryptophan-auxotrophic E. coli strains to 4-fluoroindole or 5-fluoroindole as essential precursors, forcing cells to utilize these fluorinated precursors to synthesize tryptophan analogs and incorporate them into the proteome. Multi-omics analysis showed that complete adaptation to fluorinated tryptophan requires relatively few genetic mutations but is accompanied by large-scale rearrangements of cellular networks, including reconstruction of regulatory networks, changes in membrane integrity, and adjustments in protein folding quality control. These findings provide a molecular foundation for understanding cellular adaptation mechanisms to ncAAs and offer important insights for synthetic biology and biotechnology applications.

### 3.8. Application of Synthetic Biology Tools in Peptide Drug Discovery

The development of synthetic biology tools has provided new possibilities for peptide drug discovery. By constructing efficient heterologous expression pathways, recombinant peptides containing ncAAs can be produced [59,60]. As mentioned earlier, the in vivo biosynthesis platform developed by Costello et al. [54] can create novel macrocyclic peptide libraries containing multiple unique ncAAs. This platform utilizes plasmid-encoded tRNA-synthetase pairs and quadruplet codon strategies to achieve biosynthesis of up to 100 novel macrocyclic peptide structures in *E. coli*. This high-throughput biosynthesis method provides a rich source of compounds for discovering new peptide drugs.

### 3.9. Saccharomyces cerevisiae as a Eukaryotic Expression System

*Saccharomyces cerevisiae*, as a eukaryotic expression system, offers unique advantages in producing complex peptides and proteins. Compared with *E. coli*, yeast possesses more complete protein folding and modification systems, enabling proper folding of peptides with complex three-dimensional structures and appropriate post-translational modifications. Genetic code expansion technology has been successfully applied in *Saccharomyces cerevisiae* to achieve ncAA incorporation [61,62]. This eukaryotic expression system is particularly suitable for producing complex peptide drugs requiring eukaryotic cell modifications, such as glycosylated peptides or peptides containing disulfide bonds.

### 3.10. Mining Protease-Resistant Peptides from Microbiomes

The microbiome represents a vast source of natural products containing numerous bioactive peptides. Some microorganisms have evolved mechanisms to resist their own proteases, which can provide inspiration for designing human protease-resistant peptides. Through metagenomics and bioinformatics approaches, novel protease-resistant peptides can be mined from microbiomes [63,64]. Synthetic biology methods enable heterologous expression and structural optimization of these natural products.

## 4. Clinical Translation and Emerging Applications

**Figure 5:**
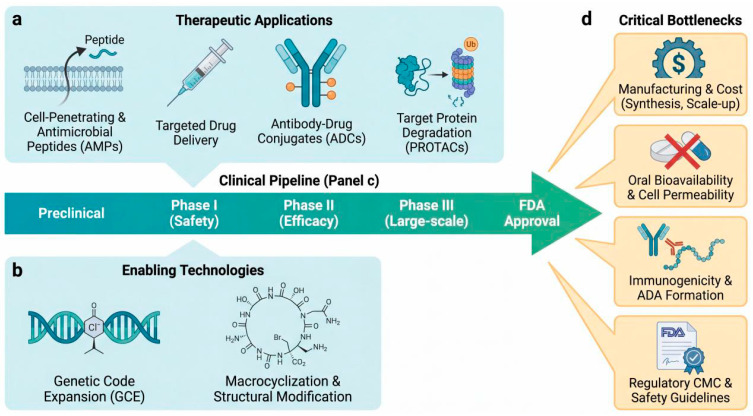
Clinical translation landscape and implementation hurdles for non-canonical amino acid (ncAA)-based therapeutics. Schematic overview of the strategic framework for transitioning ncAA-modified peptides from laboratory discovery to clinical application. (**a**) Primary therapeutic application areas utilizing ncAA modifications, including cell-penetrating and antimicrobial peptides (AMPs), targeted drug delivery vehicles, site-specific antibody–drug conjugates (ADCs), and target protein degradation via proteolysis-targeting chimeras (PROTACs). (**b**) Enabling chemical and biological technologies, such as genetic code expansion (GCE) and macrocyclization, utilized to optimize peptide drug-like properties. (**c**) The standard clinical development pipeline, progressing from preclinical evaluation through Phase I (safety), Phase II (efficacy), and Phase III (large-scale) trials to final FDA regulatory approval. (**d**) Critical bottlenecks and translational hurdles limiting clinical adoption. These challenges encompass manufacturing scaling and high synthesis costs, pharmacokinetic barriers such as poor oral bioavailability and cell permeability, safety concerns regarding unpredictable immunogenicity and anti-drug antibody (ADA) formation, and the establishment of rigorous regulatory chemistry, manufacturing, and controls (CMC) guidelines.

The structural and functional versatility afforded by ncAAs has fundamentally redefined the pharmacological potential of peptide-based drugs. By surmounting the inherent limitations of natural peptides—specifically their rapid clearance and proteolytic fragility—ncAA-modified scaffolds have enabled the development of highly stable and selective therapeutic modalities, ranging from targeted antimicrobial peptides to sophisticated ADCs and PROTACs. Despite these biological advantages, the transition from laboratory-scale synthesis to clinical approval requires overcoming a unique set of translational hurdles. These include the optimization of large-scale manufacturing processes, the assessment of long-term immunogenicity, and the navigation of regulatory pathways for novel chemical entities that do not conform to standard biological definitions. As the clinical pipeline for these molecules matures from preclinical assessment toward late-stage trials, addressing these industrial and pharmacokinetic bottlenecks will be essential for realizing the next generation of precision medicines (Figure 5).

### 4.1. Anti-Degradation Engineering of Antimicrobial Peptides

Antimicrobial peptides (AMPs) are important components of innate immunity with broad-spectrum antimicrobial activity. However, the in vivo stability issues of antimicrobial peptides have severely limited their clinical applications [65,66]. D-amino acid modification has proven to be an effective strategy for enhancing antimicrobial peptide stability. Similarly, the incorporation of D-amino acids in ultra-short lipopeptides (such as Lip7) led to a 2- to 32-fold increase in half-life when exposed to digestive proteases (pepsin, trypsin, and cathepsin K), maintaining therapeutic efficacy in in vivo mouse infection models [67,68].

### 4.2. Site-Specific Conjugation of Antibody–Drug Conjugates (ADCs)

Although full-length antibodies are large proteins, the design of next-generation antibody–drug conjugates (ADCs) increasingly relies on protease-resistant peptide linkers and peptide-based targeting homing ligands [69]. Chemical or biological incorporation of ncAAs into these peptide domains allows for precise site-specific conjugation and resistance against systemic proteases, preventing premature drug release in circulation [21].

### 4.3. PROTAC and Targeted Protein Degradation Technology

Proteolysis-targeting chimeras (PROTACs) are technologies that selectively degrade target proteins by utilizing the ubiquitin–proteasome system. PROTAC molecules typically consist of a target protein ligand, an E3 ubiquitin ligase ligand, and a linker [70,71]. Zheng et al. [22] developed a genetic code expansion-based intracellular approach to evaluate E3 ligases for targeted protein degradation. This method can express E3 ligase variants containing tetrazine-bearing ncAAs in living cells and conjugate them to substrate-binding proteins via click chemistry. These E3 ligase-binder constructs can be used to evaluate target protein degradation efficiency. This ligand-free degrader (ELF degrader) platform preserves the native state of E3 ligases, enables probing of any E3 surface region in live cells, and is applicable to a broad range of E3 ligases. This approach provides a versatile method for defining functional degron sites, guiding degrader design, and unlocking new E3 ligases without known ligands for therapeutic applications.

### 4.4. Protease-Activated Prodrug Design

Protease-activated prodrugs are strategies that utilize proteases specifically highly expressed in the tumor microenvironment to activate drugs [72]. The introduction of ncAAs can design more precise protease-responsive linkers. Goettig et al. [4] noted that many ncAAs confer protease resistance to peptides, making them potential protease inhibitors and tools for substrate optimization. Other applications include prodrug design, where drugs are linked to ncAA carriers via protease-cleavable linkers and specifically activated at tumor sites.

### 4.5. Clinical Trial Progress and Challenges

Although ncAA-modified peptide drugs have demonstrated tremendous potential in preclinical research—particularly in overcoming the inherent instability of natural peptides—the translation of these engineered molecules into clinically approved therapeutics remains a complex endeavor. Current clinical trials are diversifying, with a strong focus on oncology (stapled peptides), infectious diseases (antimicrobial peptidomimetics), and targeted delivery (Table 2). The integration of ncAAs fundamentally alters the physicochemical properties of the peptide, which, while beneficial for efficacy, introduces a unique set of translational bottlenecks. The primary challenges include:

1. Production Costs and Scalability: The synthesis of ncAAs often requires complex, multi-step asymmetric chemistry with low overall yields. Integrating these residues into peptides via solid-phase peptide synthesis (SPPS) or emerging biological platforms (e.g., genetic code expansion) presents significant technical difficulties at an industrial scale. Maintaining batch-to-batch consistency and high enantiomeric purity substantially elevates the cost of goods (COGs) [73].
2. Pharmacokinetics and Bioavailability: While the incorporation of ncAAs (such as D-amino acids or α-methylated residues) successfully confers resistance against endogenous proteases and peptidases, oral bioavailability remains a formidable hurdle [74]. Due to their molecular weight and hydrophilicity, most ncAA-peptides still require subcutaneous or intravenous administration. Strategies to enhance cell permeability and oral absorption, such as hydrophobic tagging and macrocyclization, are still undergoing clinical validation.
3. Immunogenicity and Toxicity: The human immune system is calibrated to recognize canonical biological structures [75,76]. The introduction of unnatural structural motifs, particularly large bio-orthogonal tags or highly fluorinated side chains, risks creating novel epitopes. This can trigger the formation of anti-drug antibodies (ADAs), which may neutralize the therapeutic effect or cause hypersensitivity reactions, necessitating rigorous and prolonged safety evaluations.
4. Regulatory Challenges: The regulatory landscape for ncAA-peptides bridges the gap between small molecules and biologics. Regulatory agencies (such as the FDA and EMA) face challenges in standardizing the approval pathways for these novel chemical entities (NCEs) [77]. Defining acceptable limits for synthetic impurities, establishing standardized quality control metrics for biological manufacturing of ncAA-peptides, and predicting long-term off-target effects require continuous and unprecedented dialogue between sponsors and regulators.

**Table 2:** Representative ncAA-containing Peptide Drugs in Clinical Development.

Drug Candidate	Target/Indication	Key ncAA Modification(s)	Current Clinical Status	Sponsor/Company
Tirzepatide [78]	GLP-1/GIP Receptors (Type 2 Diabetes, Obesity)	Aib (αα-aminoisobutyric acid) at position 2 to prevent DPP-4 cleavage.	Approved (Phase IV ongoing)	Eli Lilly
ALRN-6924 [79]	p53-MDMX/MDM2 Antagonist (Solid tumors, Lymphoma)	Olefinic ncAAs (α,αα,α-disubstituted) forming a hydrocarbon staple.	Phase I/II	Aileron Therapeutics
Murepavadin (POL7080) [80]	LptD targeting (Pseudomonas aeruginosa pneumonia)	D-amino acids, L-t-butylglycine within a cyclic β-hairpin scaffold.	Phase III (IV)/Phase I (Inhaled)	Polyphor/Spexis
Abaloparatide [81]	PTH1 Receptor (Osteoporosis)	Aib (αα-aminoisobutyric acid) substitution to stabilize αα-helical structure.	Approved	Radius Health
Nemifitide [82]	Melanocortin analog (Major Depressive Disorder)	4-Fluoro-phenylalanine (4-F-Phe) to enhance metabolic stability.	Phase III(Suspended/Completed)	Innapharma
Bremelanotide [83]	Melanocortin 4 Receptor (Hypoactive Sexual Desire Disorder)	D-Phenylalanine to prevent rapid proteolytic degradation.	Approved	Palatin Technologies
Cilengitide [84]	αVβ3/αVβ5 Integrins (Glioblastoma)	D-Phenylalanine within a cyclic RGD pentapeptide scaffold.	Phase III(Completed)	Merck KGaA

Abbreviations: Aib, α-aminoisobutyric acid; GLP-1, glucagon-like peptide-1; GIP, gastric inhibitory polypeptide; DPP-4, dipeptidyl peptidase-4; MDMX, mouse double minute X; MDM2, mouse double minute 2; LptD, lipopolysaccharide transport protein D; PTH1, parathyroid hormone receptor 1; 4-F-Phe, 4-fluoro-phenylalanine; RGD, arginine–glycine–aspartic acid.

## 5. Challenges and Future Directions

### 5.1. Current Technical Bottlenecks

While the integration of ncAAs represents a paradigm shift in designing protease-resistant peptides, the field must overcome several substantial technical and biological barriers before these engineered molecules can realize their full therapeutic and industrial potential (Table 3). The current bottlenecks span the entire development pipeline, from computational design to biomanufacturing and clinical translation.

#### 5.1.1. Biological and Translational Hurdles (RF1 Competition, Multiplexing Limitations)

Although genetic code expansion (GCE) technology has made remarkable strides, the incorporation efficiency of ncAAs is highly sensitive to the local mRNA sequence context and the structural environment of the ribosomal exit tunnel [85]. Furthermore, in standard amber suppression systems (using the UAG stop codon), competition with endogenous Release Factor 1 (RF1) often leads to premature translation termination and truncated peptide products, drastically reducing overall yields [86].

The technology required to simultaneously incorporate multiple different ncAAs into a single peptide scaffold is not yet fully mature. The limited availability of mutually orthogonal tRNA/aminoacyl-tRNA synthetase (aaRS) pairs, combined with a finite number of available “blank” codons (e.g., stop codons or quadruplet codons), restricts the complexity of multiplexed peptide design [87,88].

Introducing ncAAs—particularly bulky bio-orthogonal tags, highly fluorinated side chains, or D-amino acids—can unpredictably perturb the native secondary and tertiary structure of the peptide [89]. Current computational modeling and artificial intelligence (AI) tools often struggle to accurately predict the folding thermodynamics and structural consequences of these unnatural modifications, complicating rational drug design [90].

#### 5.1.2. Manufacturing and Downstream Processing (Metabolic Burden, Purification Workflows)

Transitioning from laboratory-scale proof-of-concept to industrial-scale manufacturing presents profound difficulties. Biological production via fermentation is often limited by the metabolic burden placed on the host organism and the high cost of supplementing exogenous ncAAs into the growth medium [91]. Additionally, the altered isoelectric points and hydrophobicity profiles of ncAA-peptides can complicate downstream purification, requiring custom, non-standard chromatography workflows [92].

#### 5.1.3. Clinical and Pharmacokinetic Barriers (Cell Permeability, Immunogenicity)

While ncAAs successfully shield peptides from proteolytic degradation, they do not inherently solve the issue of cellular permeability [93]. Highly modified, protease-resistant peptides frequently violate “rule of five” parameters (due to high molecular weight and topological polar surface area), resulting in poor oral bioavailability and an inability to hit intracellular targets without the aid of complex delivery vehicles [94].

The long-term in vivo safety, metabolic clearance, and exact physiological fate of ncAAs are not entirely understood. Because ncAAs are completely foreign to normal human metabolism, there is an elevated risk that their degradation byproducts may be toxic, or that the highly modified peptides may trigger an unwanted adaptive immune response (immunogenicity) upon repeated dosing [95].

### 5.2. Future Development Directions

**Figure 6:**
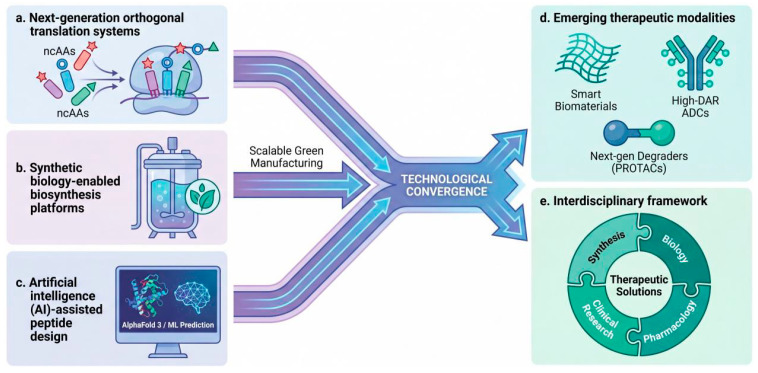
Future roadmap for the development and clinical translation of ncAA-engineered peptides. Schematic representation of the converging technological frontiers required to advance non-canonical amino acid (ncAA) technologies from molecular design to clinical implementation. (**a**) Next-generation orthogonal translation systems. Engineered orthogonal ribosomes and specialized tRNA–synthetase pairs facilitate the simultaneous incorporation of multiple distinct ncAAs, enabling the synthesis of multifunctional and hyper-stable peptide architectures. (**b**) Synthetic biology-enabled biosynthesis platforms. Engineered microbial cell factories and optimized metabolic pathways allow for the sustainable, industrial-scale green manufacturing of complex ncAAs and peptides to reduce production costs. (**c**) Artificial intelligence (AI)-assisted peptide design. Machine learning models (such as AlphaFold 3) trained on structural-stability datasets enable the in silicoprediction of optimized pharmacokinetic profiles, which can be validated via high-throughput screening. (**d**) Emerging therapeutic modalities. Technological convergence enables advanced clinical platforms, including programmable smart biomaterials, high-precision antibody–drug conjugates (ADCs), and next-generation target degraders (PROTACs). (**e**) Interdisciplinary framework. Successful translation relies on continuous collaboration across chemical synthesis, biology, pharmacology, and clinical research to bridge the gap between basic molecular engineering and practical therapeutic solutions.

Despite significant progress in the incorporation of ncAAs for enhancing peptide stability, the field is still at an early stage of translating these technologies into clinically viable therapeutics. Future research is expected to focus on several key directions that integrate synthetic biology, metabolic engineering, and peptide drug discovery (Figure 6).

#### 5.2.1. Next-Generation Orthogonal Translation Systems

The development of next-generation orthogonal translation systems will further expand the repertoire of ncAAs that can be incorporated into peptides and proteins. Engineering more efficient tRNA–synthetase pairs and orthogonal ribosomes will allow the simultaneous incorporation of multiple ncAAs, enabling the creation of highly complex peptide architectures with enhanced stability, specificity, and biological activity.

#### 5.2.2. Synthetic Biology-Enabled Biosynthesis Factories

Synthetic biology-enabled biosynthesis platforms are expected to play a critical role in scalable production of ncAAs and ncAA-containing peptides. Advances in metabolic engineering and microbial cell factories may enable sustainable biosynthesis of fluorinated amino acids, β-amino acids, and other structurally complex ncAAs, reducing reliance on costly chemical synthesis and facilitating industrial-scale production.

#### 5.2.3. AI-Guided In Silico Peptide Design

The integration of artificial intelligence–assisted peptide design with ncAA incorporation strategies represents a promising frontier. Machine learning models trained on structural and stability data may enable predictive design of protease-resistant peptides with optimized pharmacokinetic properties. Combining computational design with high-throughput screening platforms such as mRNA display could significantly accelerate the discovery of novel peptide therapeutics.

ncAA technologies may unlock new opportunities in next-generation peptide therapeutics, including targeted protein degradation (PROTAC-based systems), ADCs, and programmable peptide biomaterials. Site-specific incorporation of ncAAs with bio-orthogonal functional groups could enable precise conjugation strategies and multifunctional therapeutic platforms.

#### 5.2.4. Interdisciplinary Clinical Translation

Future progress will rely heavily on interdisciplinary collaboration among synthetic biologists, chemists, pharmacologists, and clinicians. Bridging advances in ncAA chemistry with synthetic biology tools and clinical translation pipelines will be essential for transforming ncAA-engineered peptides from promising molecular designs into practical therapeutic solutions.

## 6. Conclusions

The integration of non-canonical amino acids (ncAAs) into peptide therapeutics represents a paradigm shift in overcoming the historical limitations of poor proteolytic stability and short in vivo half-lives. By strategically exploiting side-chain steric shielding, backbone conformational constraint, and stereochemical evasion of proteases, molecular engineers can now design peptides with unprecedented structural integrity. Concurrently, biosynthetic breakthroughs—led by genetic code expansion (GCE) platforms, orthogonal translation machineries, and host metabolic engineering for de novo precursor synthesis—are transitioning these complex molecules from expensive chemical syntheses to scalable, host-based microbial cell factories.

While significant bottlenecks remain in clinical translation, manufacturing scalability, and immunogenicity prediction, the convergence of synthetic biology with AI-guided in silico peptide design is poised to accelerate the clinical pipeline. Ultimately, ncAA-engineered peptides bridge the gap between small-molecule precision and antibody-like potency, paving the way for next-generation, hyper-stable therapeutics.

## Figures and Tables

**Table 1:** ncAA Stabilization Strategies.

ncAA Class	Representative ncAAs	Core Structural Features	Primary Stabilization Mechanism	Target Proteases Evaded	Direct Engineering Strategy & Application
Fluorinated Amino Acids [8,9,10,11,12,13,14]	• 4-Fluorotryptophan (4-F-Trp) • 5-Fluorotryptophan (5-F-Trp) • 4-Fluorophenylalanine (4-F-Phe) • Trifluoromethionine (TFM)	Replacement of C–H bonds with highly electronegative, small-radius C–F bonds. Alters side-chain polarity and electrostatic potential without major steric distortion.	Alters electron density of the scissile amide bond, reducing protease nucleophilic attack; increases local hydrophobicity and enhances hydrophobic packing.	• Chymotrypsin • Pepsin • Trypsin	Site-specific substitution at P1/P1’ positions to disrupt electrostatic complementarity with protease binding pockets (e.g., stabilizing insulin and GLP-1 analogs).
Bio-orthogonal Amino Acids [15,16,17,18,19,20,21,22]	• Nε-Azidonorleucine (Aha) • p-Azidophenylalanine (pAzF) • Propargylglycine (Pra) • Nε-propargyloxycarbonyl-L-lysine (ProcK)	Side chains containing bio-orthogonal reactive handles (azides, alkynes, alkenes, or tetrazines) that are chemically inert in vivo.	Enables site-specific, post-translational cyclization (e.g., click-chemistry triazole stapling) to restrict conformational freedom and shield scissile bonds.	• Trypsin • Chymotrypsin • Serum proteases	Incorporate azide/alkyne pairs at i, i + 4 or i, i + 7 positions, followed by click-mediated macrocyclization to yield cell-permeable, hyper-stable stapled peptides.
α-Methylated Amino Acids [23,24,25,26]	• α-Aminoisobutyric acid (Aib) • α-Methylalanine (α-Me-Ala) • α-Methylleucine (α-Me-Leu)	Substitution of the α-hydrogen atom with a methyl group, introducing a quaternary carbon center at the α-position.	Restricts Ramachandran dihedral angles (φ, ψ) to helical regions, locking the peptide into stable α- or 3_10-helices that cannot unfold to fit protease active sites.	• Elastase • Carboxypeptidase A/B • Chymotrypsin	Insert at i, i + 3 or i, i + 4 positions within helical peptide therapeutics (e.g., GLP-1 agonists, antimicrobial peptides) to enforce helicity and prevent endopeptidase cleavage.
Hydrophobic/Bulky ncAAs [27]	• Homophenylalanine (hPhe) • 1-Naphthylalanine (1-Nal) • 2-Naphthylalanine (2-Nal) • Cyclohexylalanine (Cha)	Aliphatic or aromatic side chains with significantly larger steric volume and hydrophobicity than natural counterparts (e.g., Phe, Tyr).	Creates severe steric hindrance at the protease binding cleft, physically blocking catalytic residues from approaching the peptide backbone.	• Chymotrypsin • Thrombin • Trypsin	Substitute bulky hydrophobic ncAAs at or adjacent to known cleavage hotspots (P1 or P1’ positions) to physically shield vulnerable amide bonds while enhancing receptor binding.
D-Amino Acids [28,29,30,31]	• D-Alanine (D-Ala) • D-Phenylalanine (D-Phe) • D-Leucine (D-Leu)	Inversion of stereochemical configuration at the α-carbon from the natural L-configuration to the D-configuration.	Stereochemical mismatch with the L-stereospecific binding clefts of mammalian proteases, preventing proper substrate alignment and hydrogen-bonding networks.	• Trypsin • Chymotrypsin • Pepsin • Aminopeptidases	Substitute D-amino acids at N- or C-termini to block exopeptidases, or employ full retro-inverso design (reversing sequence and chirality) to yield protease-immune peptidomimetics.
β-Amino Acids [32,33]	• β3-Alanine (β3-Ala) • β2-Phenylalanine (β2-Phe) • Cyclic β-amino acids (ACPC, APC)	Insertion of an additional methylene group (–CH2–) between the α-carbon and carbonyl carbon in the peptide backbone.	Alters backbone geometry, spacing, and hydrogen-bonding alignment, rendering the scissile bond completely unrecognizable to L-α-specific proteases.	• Trypsin • Proteinase K • Pronase • Pepsin	Implement systematic α-to-β substitution (backbone foldamers) in active segments to create stable helical folds (e.g., 12- or 14-helices) while maintaining side-chain topography.
Bio-orthogonal Amino Acids [15,16,17,18,19,20,21,22]	• Nε-Azidonorleucine (Aha) • p-Azidophenylalanine (pAzF) • Propargylglycine (Pra) • Nε-propargyloxycarbonyl-L-lysine (ProcK)	Side chains containing bio-orthogonal reactive handles (azides, alkynes, alkenes, or tetrazines) that are chemically inert in vivo.	Enables site-specific, post-translational cyclization (e.g., click-chemistry triazole stapling) to restrict conformational freedom and shield scissile bonds.	• Trypsin • Chymotrypsin • Serum proteases	Incorporate azide/alkyne pairs at i, i + 4 or i, i + 7 positions, followed by click-mediated macrocyclization to yield cell-permeable, hyper-stable stapled peptides.

**Table 3:** Key Technical Bottlenecks in the Design and Production of ncAA-Peptides.

Bottleneck Category	Specific Challenge	Impact on Peptide Development	Emerging/Potential Solutions
Genetic Engineering & Translation	RF1 competition & context bias	Low overall yield; high percentage of truncated byproducts.	Genomically recoded organisms (e.g., RF1-knockout strains); evolved orthogonal ribosomes.
Multiplexing	Lack of mutually orthogonal pairs; limited codons	Restricts the design of highly complex, multifunctional peptides.	Quadruplet codon decoding; synthetic genome synthesis; orthogonal translation networks.
Computational Design	Conformational perturbation	High failure rate in rational design; loss of target binding affinity.	Advanced machine learning (AlphaFold 3/Rosetta) trained specifically on ncAA-peptide datasets.
Manufacturing & Cost	Metabolic burden; expensive raw materials	Prohibitive Cost of Goods (COGs); challenges in industrial scale-up.	De novo metabolic engineering (cell factories synthesizing ncAAs directly from glucose).
Pharmacokinetics	Poor cell membrane permeability	Prevents targeting of intracellular proteins; requires injection.	Cyclization strategies; hydrophobic tagging; integration with nanoparticle delivery systems.
Clinical Safety	Unpredictable immunogenicity	Risk of anti-drug antibodies (ADAs); potential toxicity of metabolites.	Rigorous in silico epitope prediction; long-term in vivo mammalian safety studies.

Abbreviations: RF1, release factor 1; ncAA, non-canonical amino acid; GCE, genetic code expansion; COGs, cost of goods; ADAs, anti-drug antibodies.

## Data Availability

Data sharing not applicable No new data were created or analyzed in this study. Data sharing is not applicable to this artic.

## Abbreviations

1. Core Concepts & Methodologies	
ncAA/ncAAs	Non-canonical Amino Acids;
GCE	Genetic Code Expansion;
SPPS	Solid-Phase Peptide Synthesis;
aaRS/RS	Aminoacyl-tRNA Synthetase;
RF1	Release Factor 1;
BEAR	Backbone Extension Acyl Rearrangement;
IVT	In Vitro Translation;
CMC	Chemistry, Manufacturing, and Controls.
2. Chemical Structures, Modifications & ncAAs	
Aib	α-Aminoisobutyric Acid;
β-Nal	β-Naphthylalanine;
pFF	para-Fluorophenylalanine;
oFF	ortho-Fluorophenylalanine;
4-F-Phe	4-Fluoro-phenylalanine;
4-F-Trp	4-Fluorotryptophan;
5-F-Trp	5-Fluorotryptophan;
Aha	Azidohomoalanine;
Hpg	Homopropargylglycine;
Nϵ-AzK	Nϵ-Azido-L-lysine;
CNpyrA	Cyanopyridylalanine;
pyr-thn	Pyridine-thiazoline;
pGlu	Pyroglutamic Acid;
Ac	Acetyl;
NH_2_	Amide/Amidation.
3. Therapeutic Modalities & Biology	
AMP/AMPs	Antimicrobial Peptides;
ADC/ADCs	Antibody–Drug Conjugates;
PROTAC/PROTACs	Proteolysis-Targeting Chimeras;
TPD	Targeted Protein Degradation;
DAR	Drug-to-Antibody Ratio;
GLP-1	Glucagon-Like Peptide-1;
GIP	Gastric Inhibitory Polypeptide;
DPP-4	Dipeptidyl Peptidase-4;
MDM2	Mouse Double Minute 2;
MDMX	Mouse Double Minute X;
LptD	Lipopolysaccharide Transport Protein D;
PTH1	Parathyroid Hormone Receptor 1;
RGD	Arginine–Glycine–Aspartic Acid;
MRSA	Methicillin-Resistant Staphylococcus aureus;
USP15	Ubiquitin-Specific Protease 15.
4. Metabolic Precursors, Analysis & Others	
PEP	Phosphoenolpyruvate;
E4P	Erythrose 4-Phosphate;
PYR	Pyruvate;
Trp	Tryptophan;
TrpB/TrpCBA	Tryptophan Synthase Subunits;
ADA/ADAs	Anti-Drug Antibodies;
COGs	Cost of Goods;
NCE/NCEs	Novel Chemical Entities;
PK	Pharmacokinetics;
AI	Artificial Intelligence;
ML	Machine Learning;
NMR	Nuclear Magnetic Resonance;
HPLC	High-Performance Liquid Chromatography;
HELM	Hierarchical Editing Language for Macromolecules;
FDA	Food and Drug Administration;
EMA	European Medicines Agency;
DPI	Dots Per Inch.
